# A New Erythrinan Alkaloid Glycoside from the Seeds of *Erythrina crista-galli*

**DOI:** 10.3390/molecules22091558

**Published:** 2017-09-16

**Authors:** Qing-Wei Tan, Jian-Cheng Ni, Pei-Hua Fang, Qi-Jian Chen

**Affiliations:** 1 Key Laboratory of Bio-Pesticide and Chemistry-Biology, Ministry of Education, Fujian Agriculture and Forestry University, Fuzhou 350002, Fujian, China; njc2130215001@163.com (J.-C.N.); fjqzfph320@163.com (P.-H.F.); 2 Key Laboratory of Plant Virology of Fujian Province, Institute of Plant Virology, Fujian Agriculture and Forestry University, Fuzhou 350002, Fujian, China

**Keywords:** *Erythrina crista-galli*, fabaceae, Erythrinan alkaloid, tobacco mosaic virus, antiviral activity

## Abstract

A new *Erythrina* alkaloid glycoside, named erythraline-11β-*O*-glucopyranoside, was isolated from the seeds of *Erythrina crista-galli* L., together with five known *Erythrina* alkaloids and an indole alkaloid. The structure of the new alkaloid glycoside was elucidated by spectroscopic methods, and all of the compounds were evaluated for their antiviral activity against tobacco mosaic virus.

## 1. Introduction

**Figure 1:**
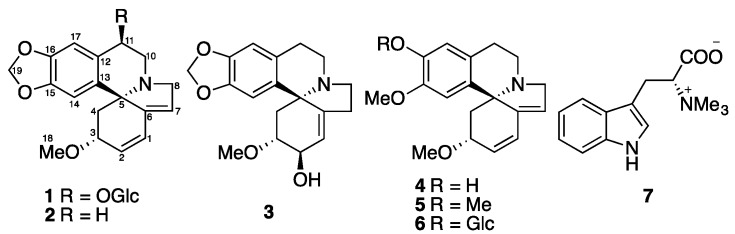
Structure of Compounds **1**–**7**.

The *Erythrina* genus, including approximately 200 species, is mainly distributed in tropical and subtropical areas. Four local and six introduced *Erythrina* species are distributed in China [1]. *Erythrina crista-galli* L. (Fabaceae), known as cockspur coral tree, is a popular ornamental plant in South America and tropical and subtropical regions of South Asia. Pharmacological investigations have demonstrated that *E. crista-galli* seed extracts possess sedative, hypertensive, laxative, and diuretic activities [2]. Phytochemical studies on this plant showed the presence of *Erythrina* [3,4] and benzylisoquinoline alkaloids [5,6]. The alkaloids of the *Erythrina* type, which possess a unique spirocyclic structure, are responsible for these activities. Great efforts have been made to reveal the biosynthetic pathway and mechanism for the *Erythrina* alkaloid biosynthesis in the past few decades [2]. Furthermore, there are chemical studies on its non-alkaloidal constituents, in which flavonoids, cinnamylphenols and pterocarpans were reported functioning as phytoalexins and possessing antimicrobial activity [7,8,9,10,11]. In this paper, we describe the isolation and structural characterization of a new *Erythrina* alkaloids glucoside, erythraline-11β-*O*-glucopyranoside (**1**), together with six known alkaloids (**2**–**7**) (Figure 1) from the seeds of *Erythrina crista-galli*, and these isolated compounds were evaluated for their inhibitory activity against tobacco mosaic virus (TMV) using the leaf-disc method.

## 2. Results and Discussion

Phytochemical study of MeOH extract of the seeds of *Erythrina crista-galli* led to the isolation of a new Erythrinan alkaloid glucoside, erythraline-11β-*O*-glucopyranoside (**1**), together with five known Erythrinan alkaloids, erythraline (**2**) [12], erythratine (**3**) [13], erysodine (**4**) [14], erysotrine (**5**) [13], (+)-16β-d-glucoerysopine (**6**) [15], and an indole alkaloid, (−)-hypaphorine (**7**) [16] (Figure 1). The known structures were identified by comparison of their spectroscopic data with those reported in the literature.

**Figure 2:**
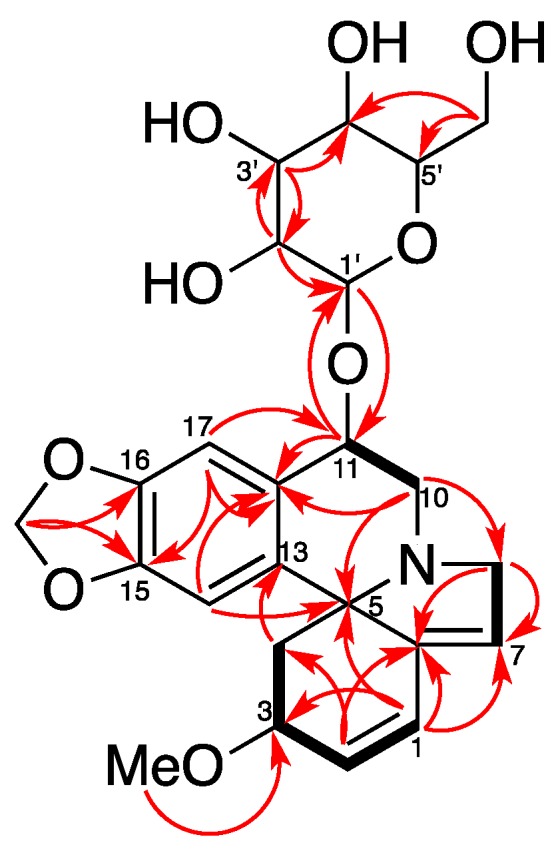
^1^H-^1^H COSY (−) and key HMBC (→) correlations of Compound **1**.

**Figure 3:**
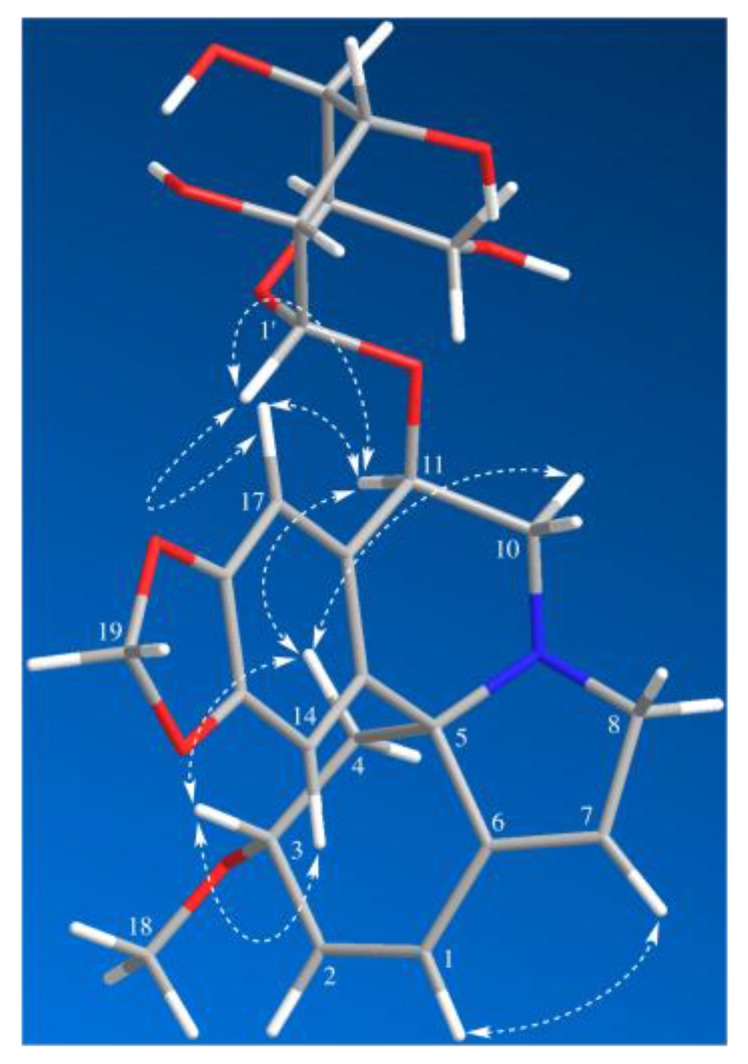
Key NOESY correlations of Compound **1**.

Compound **1** was isolated as a white amorphous powder. It was assigned with a molecular formula of C_24_H_29_NO_9_ by HR-ESI-MS at *m*/*z* = 510.1537 [M + Cl]^−^ (Calcd for C_24_H_29_ClNO_9_, 510.1536). The IR spectrum (Supplementary Materials) exhibited absorption bands due to an aromatic moiety and a conjugate olefin (1627, 1503, 1482 cm^−1^). The ^1^H-NMR and HSQC spectrum of **1** indicated the presence of a tetrasubstituted aromatic ring [*δ*_H_ 6.73 (1H, s) and 7.15 (1H, s)], a dioxy-methylene [*δ*_H_ 5.91 (1H, d, *J* = 1.2 Hz), 5.90 (1H, d, *J* = 1.2 Hz)], two olefins including a trisubstituted one [*δ*_H_ 6.59 (1H, dd, *J* = 10.2, 2.1 Hz), 6.04 (1H, d, *J* = 10.2 Hz), 5.78 (1H, br s)], three methylene groups [*δ*_H_ 3.89 (1H, br d, *J* = 15.5 Hz), 3.70 (1H, dd, *J* = 15.5, 3.0 Hz), 3.57 (1H, dd, *J* = 14.1, 4.8 Hz), 3.37–3.30 (1H, overlap), 2.46 (1H, dd, *J* = 11.5, 5.5 Hz), 1.73 (1H, dd, *J* = 11.5, 10.4 Hz)], a methoxy group [*δ*_H_ 3.34 (3H, s)], and a glucopyranosyl moiety [*δ*_H_ 4.61 (1H, d, *J* = 7.8 Hz), 3.92 (1H, dd, *J* = 11.8, 2.0 Hz), 3.73 (1H, dd, *J* = 11.8, 5.4 Hz), 3.42 (1H, t, *J* = 8.7 Hz), 3.39–3.28 (2H, overlap), 3.23 (1H, dd, *J* = 9.2, 7.8 Hz)]. Its ^13^C-NMR and DEPT spectra showed 24 carbon resonances, including one methoxy, five methylenes, twelve methines, and six quaternary carbons (including one olefinic). The ^13^C-NMR data of **1** were similar to those of erythraline (**2**), except for the additional signals for a glucopyranosyl group at *δ*_C_ 105.9 (C-1’), 78.2 (C-3’), 78.0 (C-5’), 75.4 (C-2’), 71.6 (C-4′), and 62.8 (C-6’), as well as an oxygenated methine appearing at *δ*_C_ 74.5 instead of a methylene carbon appearing at *δ*_C_ 25.3 (C-11) in that of **2**. The data above suggested a glucose moiety attached through an oxygen to C-11. The glucoside must have a β attachment, since the anomeric proton H-1′ (*δ*_H_ 4.61) exhibits a diaxial coupling (*J* = 7.8 Hz) with H-2’ (*δ*_H_ 3.23). The HMBC and ^1^H-^1^H COSY correlations (Figure 2) confirmed that **1** was a typical Erythrinan alkaloid having a characteristic spiro-carbon positioned in the center of rings A, B, and C with two olefins of ∆ ^1,2^ and ∆ ^6,7^ and a dioxy-methylene group attached to the C-15 and C-16. The presence of a glucose moiety at C-11 was further confirmed by HMBC correlation observed from the anomeric proton H-1’ to C-11. The HMBC correlation from methoxy protons H-18 (*δ*_H_ 3.32) to C-3 (*δ*_C_ 77.4) indicated that C-3 was substituted with a methoxy group. The relative configuration of **1** was determined by NOESY experiment. The observed cross-peaks (Figure 3) between H-3/H-14, H-4β, and correlations between H-4β/H-11, H-10α implied that the protons at C-3 and C-11 were placed at β- and α-orientation, respectively. Therefore, the structure of **1** was established as erythraline-11β-*O*-glucopyranoside. Biogenetic considerations on *Erythrina* alkaloids and the positive optical rotation value suggested that **1** has an *S* configuration at C-5 [6,14,17].

**Figure 4:**
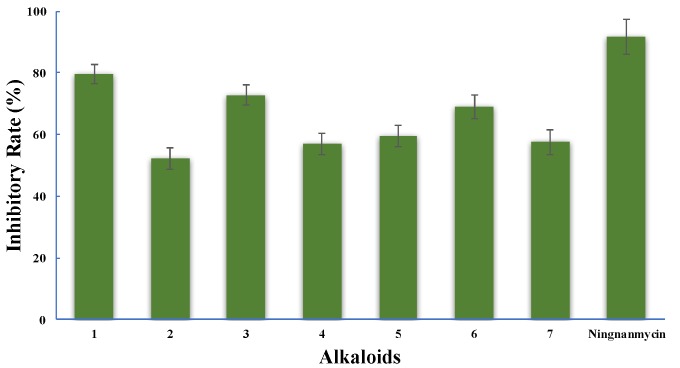
Inhibitory activity of the isolated alkaloids **1**–**7** against the replication of TMV.

Alkaloids **1**–**7** showed potent inhibition against the replication of TMV at a concentration of 500 μg/mL, with inhibitory rates varying from 52.1% to 79.7% (Figure 4). A commercial antiviral agent, ningnanmycin, exhibited 91.7% inhibition as tested under the same condition. Furthermore, the IC_50_ values of alkaloids **1**–**7** were determined as 0.59, 1.52, 1.04, 1.48, 1.28, 0.74, and 1.69 mM using the leaf-disc method, while the positive control, ningnanmycin, possessed an IC_50_ of 0.18 mM during a test under the same condition. The new alkaloid glycoside, erythraline-11β-*O*-glucopyranoside (**1**), showed a noticeable increase in the inhibition against TMV as compared with its aglycone, erythraline (**2**).

## 3. Materials and Methods

### 3.1. General Experimental Procedures

Optical rotations were measured on a JASCO-1020 polarimeter (JASCO, Tokyo, Japan). UV spectra were measured using a Perkin Elmer Lambda 800 UV/VIS spectrometer (PerkinElmer, Waltham, MA, USA). IR spectra were obtained with a Thermo Nicolet Avatar 360 FT-IR spectrometer (Thermo Nicolet Corporation, Madison, WI, USA). ^1^H- and ^13^C-NMR spectra were obtained with a Bruker AVANCE III 500 spectrometer (Bruker BioSpin, Rheinstetten, Germany) using tetramethylsilane as an internal standard. HRESIMS were obtained with a Thermo Scientific™ TSQ Quantum Access MAX triple stage quadrupole mass spectrometer (Thermo Scientific, San Jose, CA, USA). Sephadex LH–20 (25–100 μm, Pharmacia Fine Chemical Co., Ltd., Uppsala, Sweden), Lichroprep RP-18 gel (40–63 μm, Merck, Darmstade, Germany), Silica gel (200–300 mesh) and Silica gel H (Qingdao Oceanic Chemical Co., Qingdao, China) were used for column chromatography. Thin-layer chromatography (TLC) was performed on glass-backed plates coated with 0.25 mm layers of Silica gel H (Qingdao Oceanic Chemical Co., Qingdao, China). Fractions were monitored by TLC and spots were visualized by heating silica gel plates sprayed with 5% H_2_SO_4_ in EtOH. All solvents and chemicals used were of analytical reagent grade (Sinopharm Chemical Reagent Co., Ltd., Beijing, China), and water was doubly distilled before use.

### 3.2. Plant Materials

The seeds of *Erythrina crista-galli* L. were collected in July 2016 at the Fujian Agriculture and Forestry University (Fuzhou, China), and identified by Professor Chun-Mei Huang. Herbarium specimens were deposited at the Key Laboratory of Bio-Pesticide and Chemistry-Biology, Ministry of Education, Fujian Agriculture and Forestry University, Fuzhou, China (Specimen number: EC16701S).

### 3.3. Extraction and Isolation

The seeds of *E. crista-galli* L. (dried and powdered, 950 g) was extracted with MeOH (2 L, 3 d) at room temperature three times. The MeOH extracts (150 g) were dissolved in H_2_O and successively partitioned between petroleum ether, EtOAc, and n-BuOH. The EtOAc-soluble materials (7.8 g) were subjected to silica gel (200–300 mesh) column chromatography and eluted with acetone in petroleum ether (from 100:0 to 0:100) to obtained 13 fractions (Fractions 1–13). Fraction 6 (145 mg) and Fraction 7 (2.5 g) were purified with RP–18 gel column chromatography, eluting with MeOH in H_2_O (80%), and then subjected to silica gel (H, eluted with CHCl_3_/MeOH, 99:1) column chromatography, respectively, to yield Compound **2** (11.5 mg) and Compound **5** (93.8 mg). Fraction 9 (205.0 mg) was first separated with an ODS column chromatography, eluting with MeOH in H_2_O (70%), and then subjected to silica gel (H, eluted with CHCl_3_/MeOH, 98:2) column chromatography, to yield Compound **4** (35.8 mg). Fraction 11 (250 mg) was purified with RP–18 gel column chromatography, eluting with MeOH in H_2_O (60%), and then subjected to silica gel (H, eluted with CHCl_3_/MeOH, 95:5) column chromatography, to yield Compound **3** (28.1 mg). Fraction 12 (1.05 g) was purified with ODS column chromatography, eluting with MeOH in H_2_O (60%), and then subjected to silica gel (H, eluted with CHCl_3_/MeOH, 90:10) column chromatography, to yield Compound **1** (17.1 mg) and Compound **6** (23.1 mg). Fraction 13 (424 mg) was purified with ODS column chromatography, eluting with MeOH in H_2_O (40%) to yield Compound **7** (39.2 mg).

*Erythraline-11β-O-glucopyranoside* (**1**). White amorphous powder. ${\left[\alpha \right]}_{D}^{25}$ + 130.6 (*c* 0.1 MeOH); UV (MeOH) λ_max_ (log ε): 205 (1.97), 226 (0.89), 287 (0.22); IR (KBr) υ_max_: 1627, 1503, 1482 cm^−1^; HR-ESI-MS *m*/*z*: 510.1537 (Calcd for C_24_H_29_ClNO_9_, 510.1536); ^1^H-NMR (500 MHz, CD_3_OD) and ^13^C-NMR (125 MHz, CD_3_OD) data (see Table 1).

### 3.4. Anti-TMV Assay

#### 3.4.1. Virus and Host Plant

Purified TMV (strain U1) was obtained from Institute of Plant Virology, Fujian Agriculture and Forestry University, Fuzhou, Fujian, China, whose concentration was determined as 15 mg/mL using an ultraviolet spectrophotometer method ($\mathrm{virus}\;\mathrm{concentration}\;=\;(A_{260}\;\times \;\mathrm{dilution}\;\mathrm{ration})\;/{\;E}_{1\;\mathrm{cm}}^{0.1\%,\;260\;\mathrm{nm}}$). The purified virus was kept at −20 °C and was diluted to 30 μg/mL with 0.01 M PBS before use. *Nicotiana tabacum* cv. K326, which were cultivated and grown to a 5–6-leaf stage in an insect-free greenhouse, were used as an anti-TMV assay, as a systemic TMV infection host. Purified compounds were dissolved in DMSO and diluted with 0.01 M PBS to a certain concentration for test. The final concentration of DMSO in the test solution (≤2%) showed no adverse effect on the plants.

#### 3.4.2. Leaf-Disc Method

Growing leaves of *N. tabacum* cv. K326 were mechanically inoculated with TMV (30 μg/mL in 0.01 M PBS). After 6 h, leaf discs (1 cm diameter) were punched and floated on solutions for test. Discs of healthy and inoculated leaves floated on a solution of 0.01 M PBS with 2% DMSO were used as a mock and negative control, respectively. Three replicates were carried out for each sample. After incubating for 48 h at 25 °C in a culture chamber, the leaf discs were grounded in 0.01 M carbonate coating buffer (pH 9.6), and OD_405_ values were measured using TAS-ELISA method. TAS-ELISA was performed as described in the literature [18,19]. Virus concentration was calculated from a standard curve constructed using OD_405_ values of purified TMV at concentrations of 1.0, 0.5, 0.25, 0.125, and 0.0625 μg/mL. The inhibition of test solutions on TMV was calculated as follows: inhibition rate = [1 − (virus concentration of treatment) / (virus concentration of negative control)] × 100%.

## 4. Conclusions

A new *Erythrina* alkaloid glycoside together with five known *Erythrina* alkaloids and an indole alkaloid have been isolated from the MeOH extract of the seeds of *Erythrina Crista-galli* L. All the isolated alkaloids showed noticeable inhibition against the replication of TMV but in a relatively higher concentration as compared with that of the positive control agent. Further investigations are needed to reveal the biological action mechanism of *Erythrina* alkaloids.

## Figures and Tables

**Table 1:** ^1^H- and ^13^C-NMR data of Compounds **1** and **2** (*δ* in ppm, CD_3_OD)

Position	1		2	
	*δ*_C_	*δ*_H_	*δ*_C_	*δ*_H_
C-1	126.2	6.59 (1H, dd, *J* = 10.2, 2.1 Hz)	125.4	6.53 (1H, dd, *J* = 10.1, 2.3 Hz)
C-2	132.7	6.04 (1H, d, *J* = 10.2 Hz)	131.6	5.97 (1H, d, *J* = 10.1 Hz)
C-3	77.4	4.01 (1H, m)	76.2	3.96 (1H, m)
C-4	41.2	2.46 (1H, dd, *J* = 11.5, 5.5 Hz), 1.73 (1H, dd, *J* = 11.5, 10.4 Hz)	42.0	2.49 (1H, dd, *J* = 11.5, 5.5 Hz) 1.83 (1H, dd, *J* = 11.5, 10.4 Hz)
C-5	67.8	□	67.5	□
C-6	143.7	□	142.4	□
C-7	124.5	5.78 (1H, br s)	123.1	5.72 (1H, br s, 1H)
C-8	59.3	3.89 (1H, br d, *J* = 15.5 Hz) 3.70 (1H, dd, *J* = 15.5, 3.0 Hz)	57.7	3.72 (1H, dd, *J* = 14.5, 3.0 Hz) 3.53–3.45 (1H, overlap)
C-10	50.4	3.57 (1H, dd, *J* = 14.1, 4.8 Hz) 3.37–3.30 (1H, overlap)	44.6	3.53–3.45 (1H, overlap) 2.92–2.83 (1H, overlap)
C-11	74.5	4.70 (1H, t, *J* = 4.3 Hz)	25.3	2.92–2.83 (1H, overlap) 2.71–2.64 (1H, m)
C-12	129.1	□	128.1	□
C-13	132.0	□	132.7	□
C-14	106.3	6.73 (1H, s)	106.3	6.62 (1H, s)
C-15	148.9	□	146.2	□
C-16	148.1	□	145.9	□
C-17	109.7	7.15 (1H, s)	108.8	6.76 (1H, s)
C-18	56.6	3.34 (3H, s)	56.1	3.32 (3H, s)
C-19	102.3	5.91 (1H, d, *J* = 1.2 Hz) 5.90 (1H, d, *J* = 1.2 Hz)	100.8	5.90 (1H, d, *J* = 1.5 Hz) 5.87 (1H, d, *J* = 1.5 Hz)
C-1′	105.9	4.61 (1H, d, *J* = 7.8 Hz)	□	□
C-2′	75.4	3.23 (1H, dd, *J* = 9.2, 7.8 Hz)	□	□
C-3′	78.2	3.42 (1H, t, *J* = 8.8 Hz)	□	□
C-4′	71.6	3.39–3.28 (1H, overlap)	□	□
C-5′	78.0	3.39–3.28 (1H, overlap)	□	□
C-6′	62.8	3.92 (1H, dd, *J* = 11.8, 2.0 Hz) 3.73 (1H, dd, *J* = 11.8, 5.4 Hz)	□	□

## Supplementary Materials

The following are available online. The ^1^H- and ^13^C-NMR of all the isolated compounds, and the IR, UV/Vis, HRESIMS, DEPT, HSQC, HMBC, and NOESY of Compound **1**.

## References

[1] Zhang B.J., Wu B., Bao M.F., Ni L., Cai X.H. (2016). New dimeric and trimeric *Erythrina* alkaloids from *Erythrina variegata*. RSC Adv..

[2] Maier U.H., Rödl W., Deus-Neumann B., Zenk M.H. (1999). Biosynthesis of *Erythrina* alkaloids in *Erythrina crista-galli*. Phytochemistry.

[3] Ito K., Haruna M., Jinno Y., Furukawa H. (2008). Studies on the *Erythrina* alkaloids. XI. Alkaloids of *Erythrina crysta-galli*. Linn. Structure of new alkaloids, Crystamidine. Chem. Pharm. Bull..

[4] Chawla A.S., Gupta M.P., Jackson A.H. (1987). Alkaloidal constituents of *Erythrina crista-galli* flowers. J. Nat. Prod..

[5] Ju-Ichi M., Fujitani Y., Furukawa H. (1982). Structure of cristadine—A new benzylisoqunoline alkaloid. Heterocycles.

[6] Ozawa M., Kawamata S., Etoh T., Hayashi M., Komiyama K., Kishida A., Kuroda C., Ohsaki A. (2010). Structures of new *Erythrinan* alkaloids and nitric oxide production inhibitors from *Erythrina crista-galli*. Chem. Pharm. Bull..

[7] Tanaka H., Tanaka T., Etoh H. (1997). Three pterocarpans from *Erythrina crista-galli*. Phytochemistry.

[8] Mitscher L.A., Ward J.A., Drake S., Rao G.S. (1984). Antimicrobial agents from higher plants. Erycristagallin, a new pterocarpene from the roots of the bolivian coral tree, *Erythrina crista-galli*. Heterocycles.

[9] Mitscher L.A., Gollapudi S.R., Gerlach D.C., Drake S.D., Véliz E.A., Ward J.A. (1988). Erycristin, a new antimicrobial petrocarpan from *Erythrina crista-galli*. Phytochemistry.

[10] Iinuma M., Okawa Y., Tanaka T. (1994). Three new cinnamylphenols in heartwood of *Erythrina crista-galli*. Phytochemistry.

[11] Ingham J.L., Markham K.R. (1980). Identification of the Erythrina phytoalexin cristacarpin and a note on the chirality of other 6α-hydroxypterocarpans. Phytochemistry.

[12] Chawla A.S., Chunchatprasert S., Jackson A.H. (1983). Studies of *Erythrina* alkaloids: VII–^13^C-NMR spectral studies of some *Erythina* alkaloids. Org. Magn. Reson..

[13] Barton D.H., James R., Kirby G.W., Turner D.W., Widdowson D.A. (1968). Phenol oxidation and biosynthesis. Part XVIII. The structure and biosynthesis of *Erythrina* alkaloids. J. Chem. Soc. C Org..

[14] Amer M.E., El-Masry S. (1991). Three novel glycodienoid alkaloids from *Erythrina lysistemon*. J. Nat. Prod..

[15] Wanjala C.C.W., Majinda R.T. (2000). Two novel glucodienoid alkaloids from *Erythrina latissima* seeds. J. Nat. Prod..

[16] Ozawa M., Honda K., Nakai I., Kishida A., Ohsaki A. (2008). Hypaphorine, an indole alkaloid from *Erythrina velutina*, induced sleep on normal mice. Bioorg. Med. Chem. Lett..

[17] Rukachaisirikul T., Innok P., Suksamrarn A. (2008). Erythrina alkaloids and a pterocarpan from the bark of *Erythrina subumbrans*. J. Nat. Prod..

[18] Chen J., Yan X.H., Dong J.H., Sang P., Fang X., Di Y.T., Zhang Z.K., Hao X.J. (2009). Tobacco mosaic virus (TMV) inhibitors from *Picrasma quassioides* Benn. J. Agric. Food Chem..

[19] Wang Y.S., Fan H.J., Li Y., Shi Z.L., Pan Y., Lu C.P. (2007). Development of a multi-mimotope peptide as a vaccine immunogen for infectious bursal disease virus. Vaccine.

