# Supplementary material for: A New Erythrinan Alkaloid Glycoside from the Seeds of Erythrina crista-galli
Source: Molecules. 2017 Sep 16;22(9):1558. doi: 10.3390/molecules22091558 (PMC6151707; doi:10.3390/molecules22091558)
Supplement: Supplementary file 1 [file molecules-22-01558-s001.docx]

**SUPPORTING INFORMATION**

Title: A New Erythrinan Alkaloid Glycoside from the Seeds of *Erythrina crista-galli*

**Author(s):** Qing-Wei Tan *, Jian-Cheng Ni, Pei-Hua Fang and Qi-Jian Chen *

**S1. IR spectrum of erythraline-11*β*-*O*-glucopyranoside (1).**

**S2. UV/Vis spectrum of erythraline-11*β*-*O*-glucopyranoside (1).**

**S3. HRESIMS of erythraline-11*β*-*O*-glucopyranoside (1).**

**S4. ^1^H NMR spectrum (500 MHz) of erythraline-11*β*-*O*-glucopyranoside in MeOD.**

**S5. ^13^C NMR spectrum (125 MHz) of erythraline-11*β*-*O*-glucopyranoside in MeOD.**

**S6. DEPT of erythraline-11*β*-*O*-glucopyranoside in MeOD.**

**S7. ^1^H-^1^H COSY of erythraline-11*β*-*O*-glucopyranoside in MeOD.**

**S8. HSQC of erythraline-11*β*-*O*-glucopyranoside in MeOD.**

**S9. HMBC of erythraline-11*β*-*O*-glucopyranoside in MeOD.**

**S10. NOESY of erythraline-11*β*-*O*-glucopyranoside in MeOD.**

**S11. ^1^H NMR spectrum (500 MHz) of erythraline (2) in MeOD.**

**S12. ^13^C NMR spectrum (125 MHz) of erythraline (2) in MeOD.**

**S13. ^1^H NMR spectrum (500 MHz) of erythratine (3) in MeOD.**

**S14. ^13^C NMR spectrum (125 MHz) of erythratine (3) in MeOD.**

**S15. ^1^H NMR spectrum (500 MHz) of erysodine (4) in MeOD.**

**S16. ^13^C NMR spectrum (125 MHz) of erysodine (4) in MeOD.**

**S17. ^1^H NMR spectrum (500 MHz) of erysotrine (5) in MeOD.**

**S18. ^13^C NMR spectrum (125 MHz) of erysotrine (5) in MeOD.**

**S19. ^1^H NMR spectrum (500 MHz) of (+)-16*β*-D-glucoerysopine (6) in MeOD.**

**S20. ^13^C NMR spectrum (125 MHz) of (+)-16*β*-D-glucoerysopine (6) in MeOD.**

**S21. ^1^H NMR spectrum (500 MHz) of (-)-hypaphorine (7) in MeOD.**

**S22. ^13^C NMR spectrum (125 MHz) of (-)-hypaphorine (7) in MeOD.**

**S1. IR spectrum of erythraline-11*β*-*O*-glucopyranoside (1).**

**S2. UV/Vis spectrum of erythraline-11*β*-*O*-glucopyranoside (1).**

**S3. HRESIMS of erythraline-11*β*-*O*-glucopyranoside (1).**

**S4. ^1^H NMR spectrum (500 MHz) of erythraline-11*β*-*O*-glucopyranoside (1) in MeOD.**

**
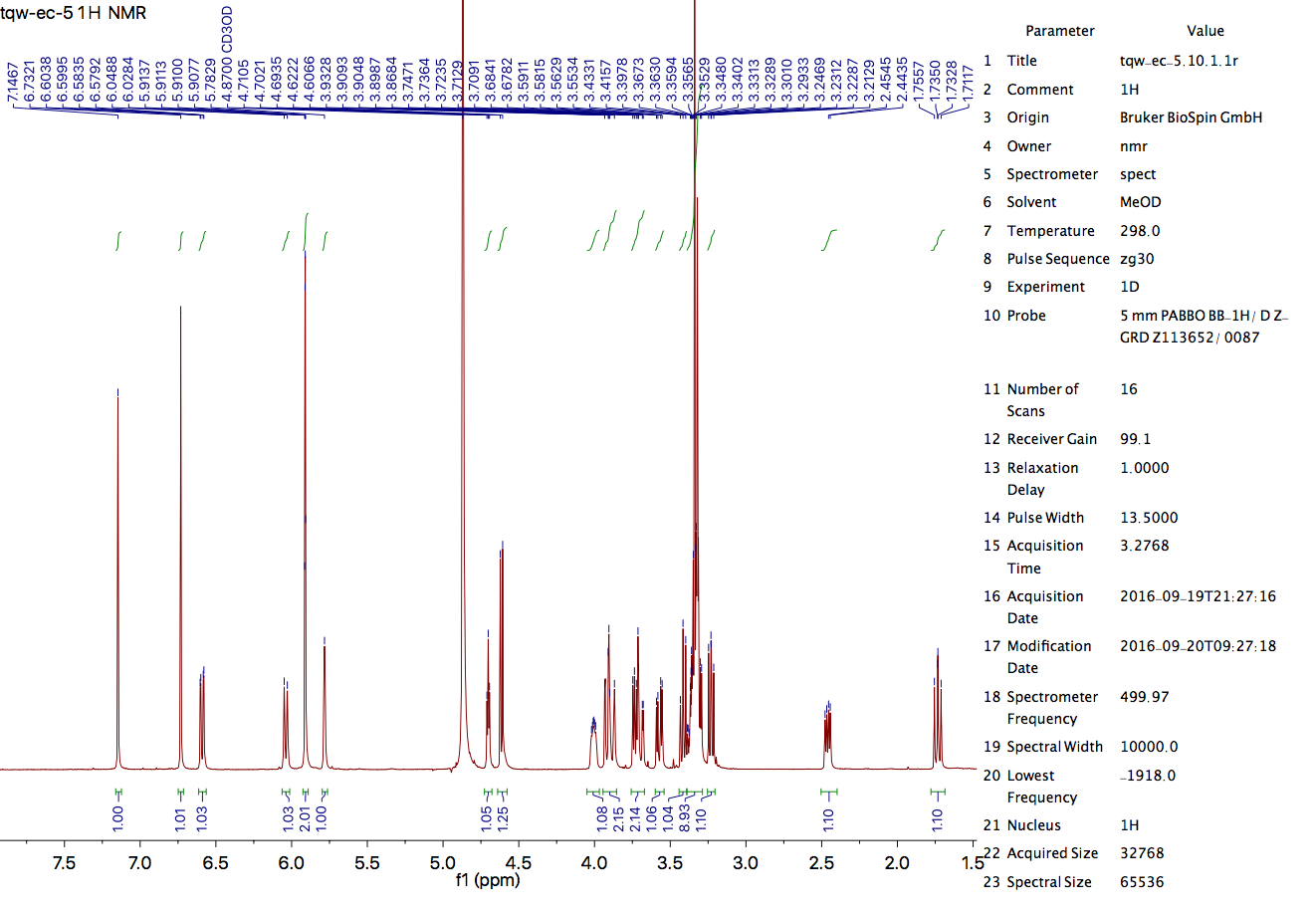
**

**S5. ^13^C NMR spectrum (125 MHz) of erythraline-11*β*-*O*-glucopyranoside (1) in MeOD.**

**
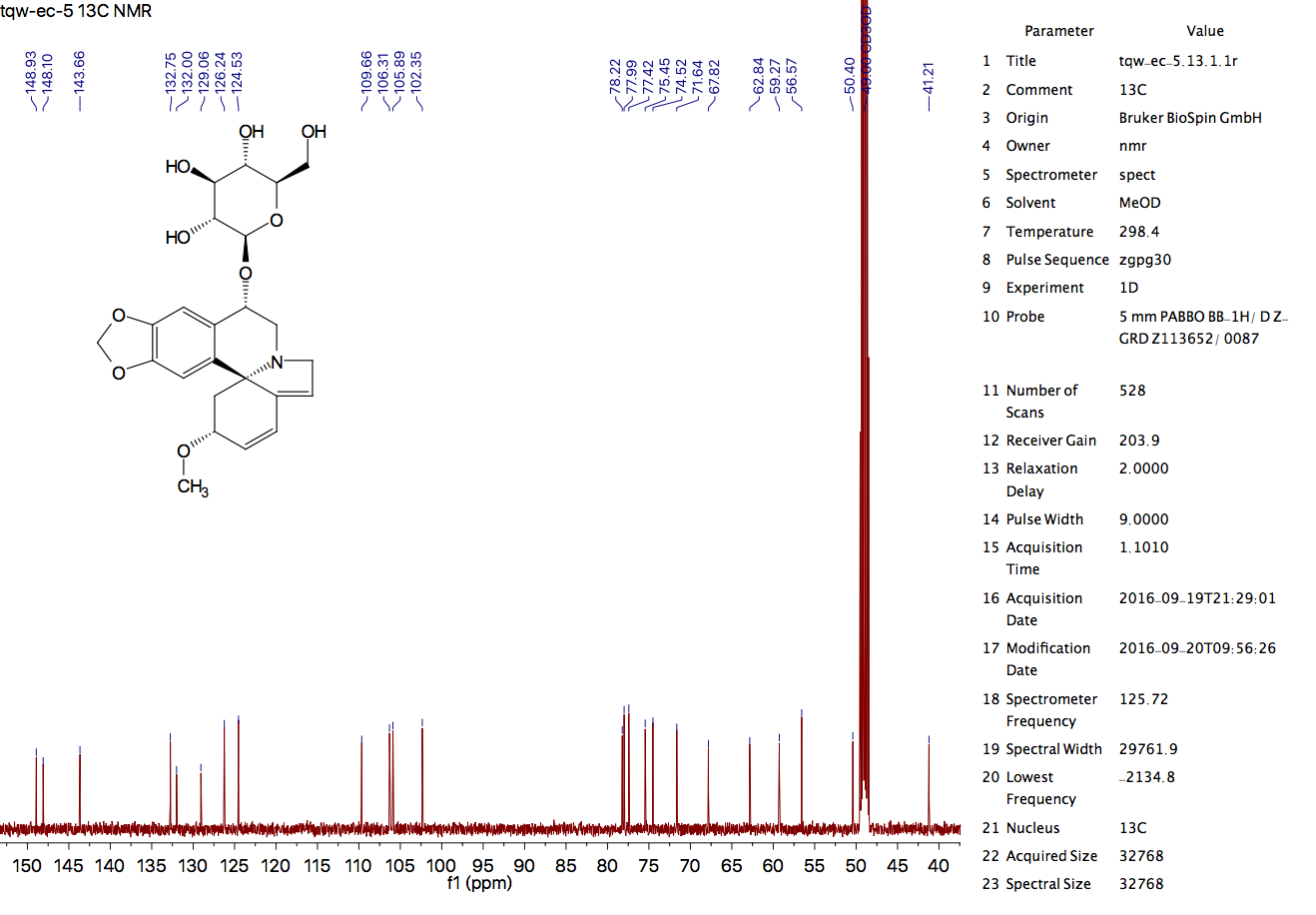
**

**S6. DEPT of erythraline-11*β*-*O*-glucopyranoside (1) in MeOD.**

**
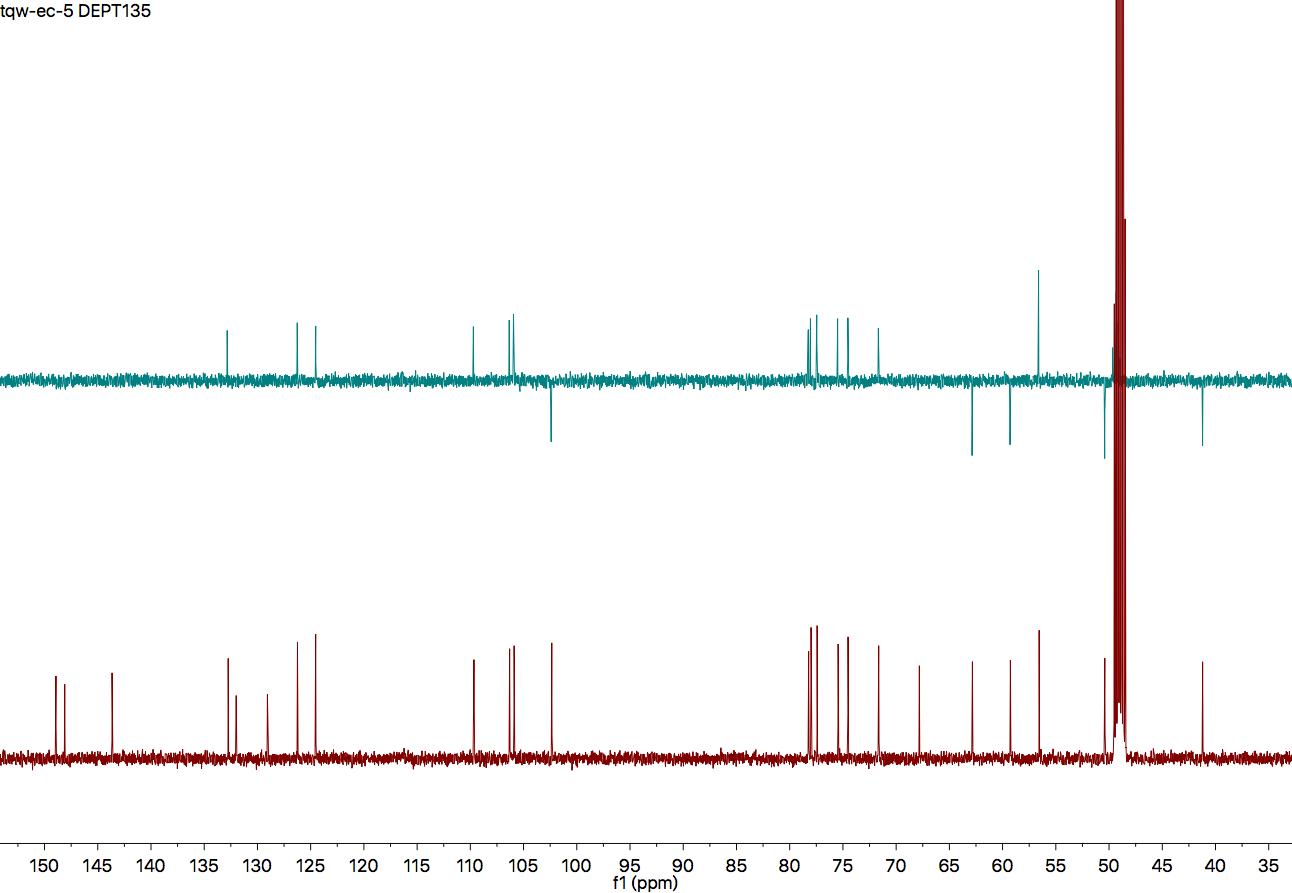
**

**S7. ^1^H-^1^H COSY of erythraline-11*β*-*O*-glucopyranoside (1) in MeOD.**

**
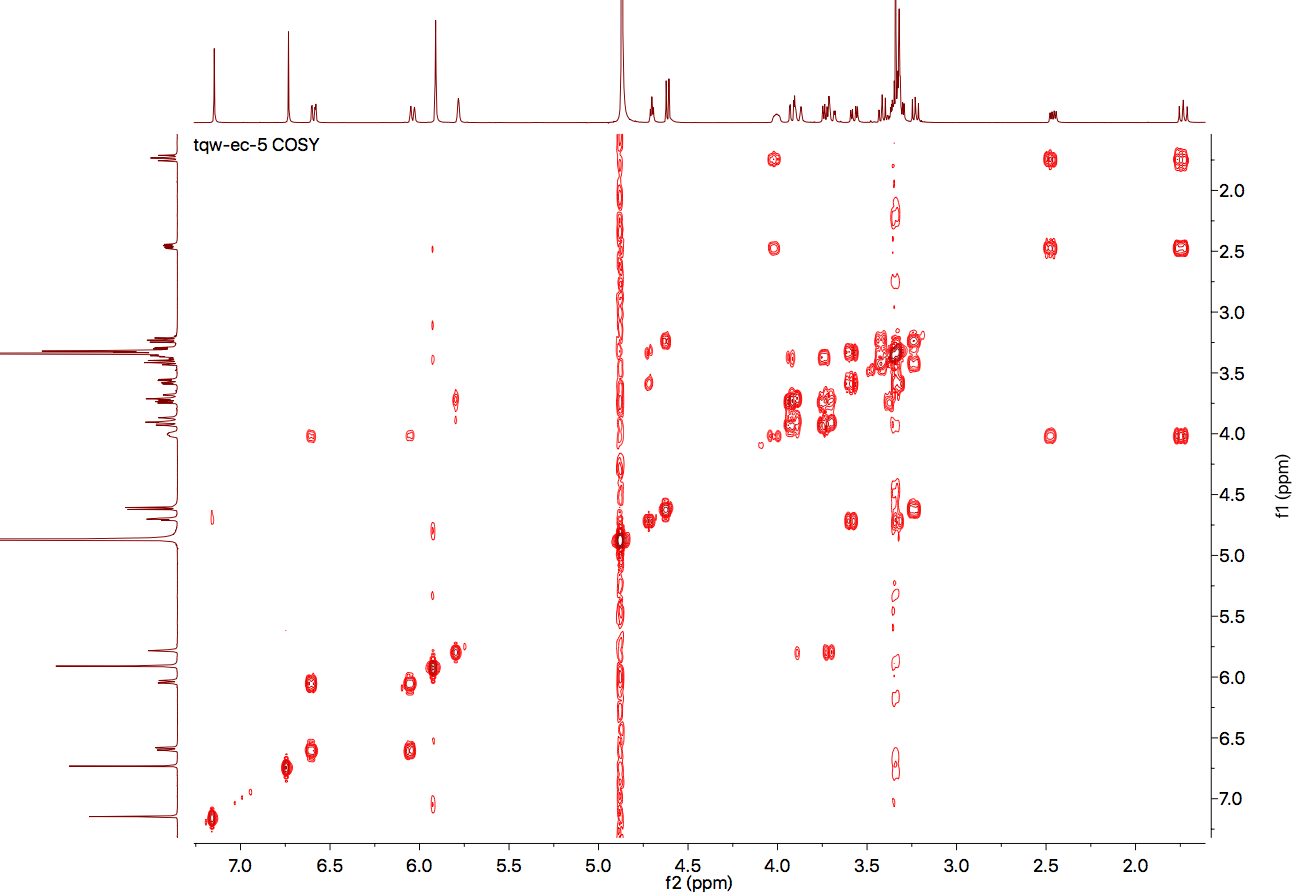
**

**S8. HSQC of erythraline-11*β*-*O*-glucopyranoside (1) in MeOD.**

**
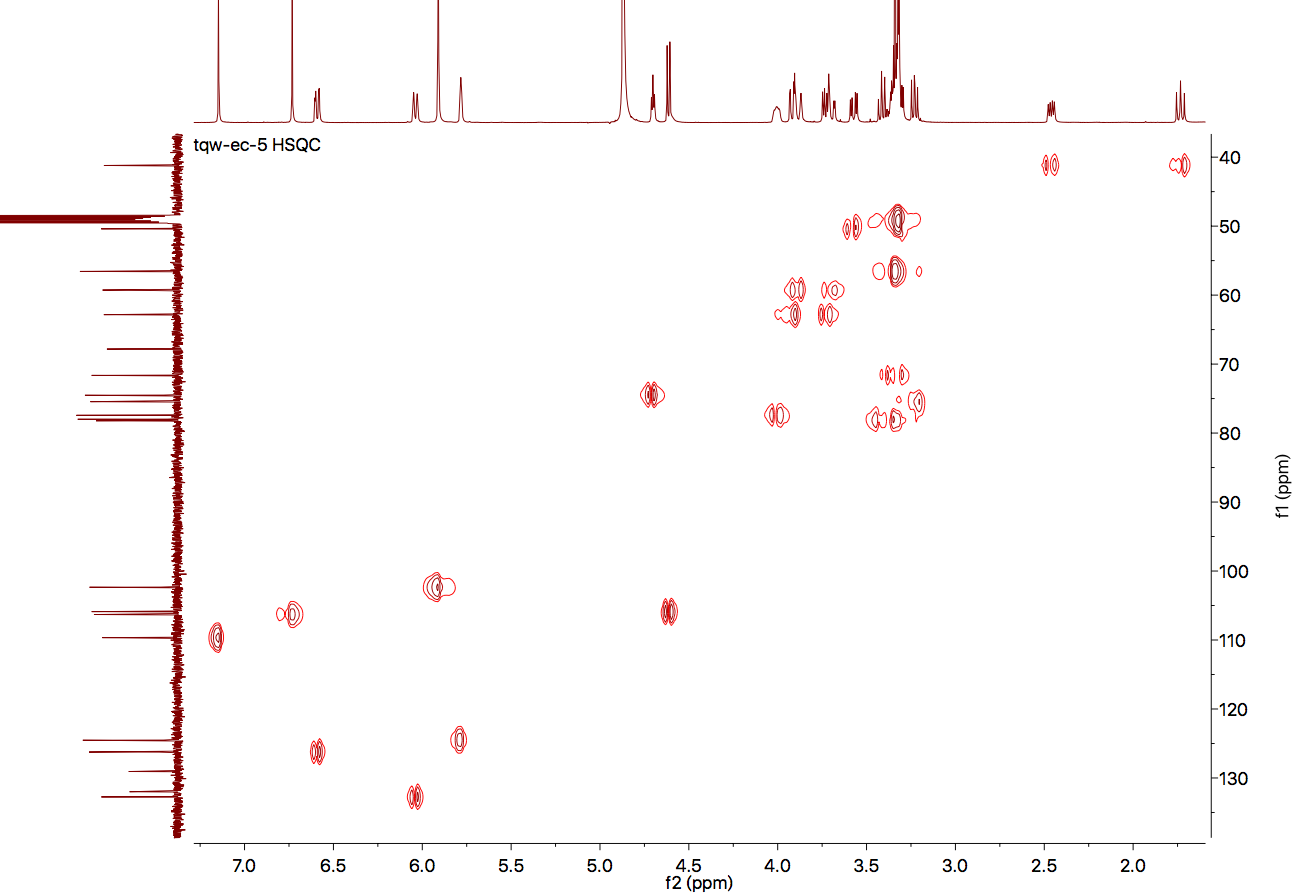
**

**S9. HMBC of erythraline-11*β*-*O*-glucopyranoside (1) in MeOD.**

**
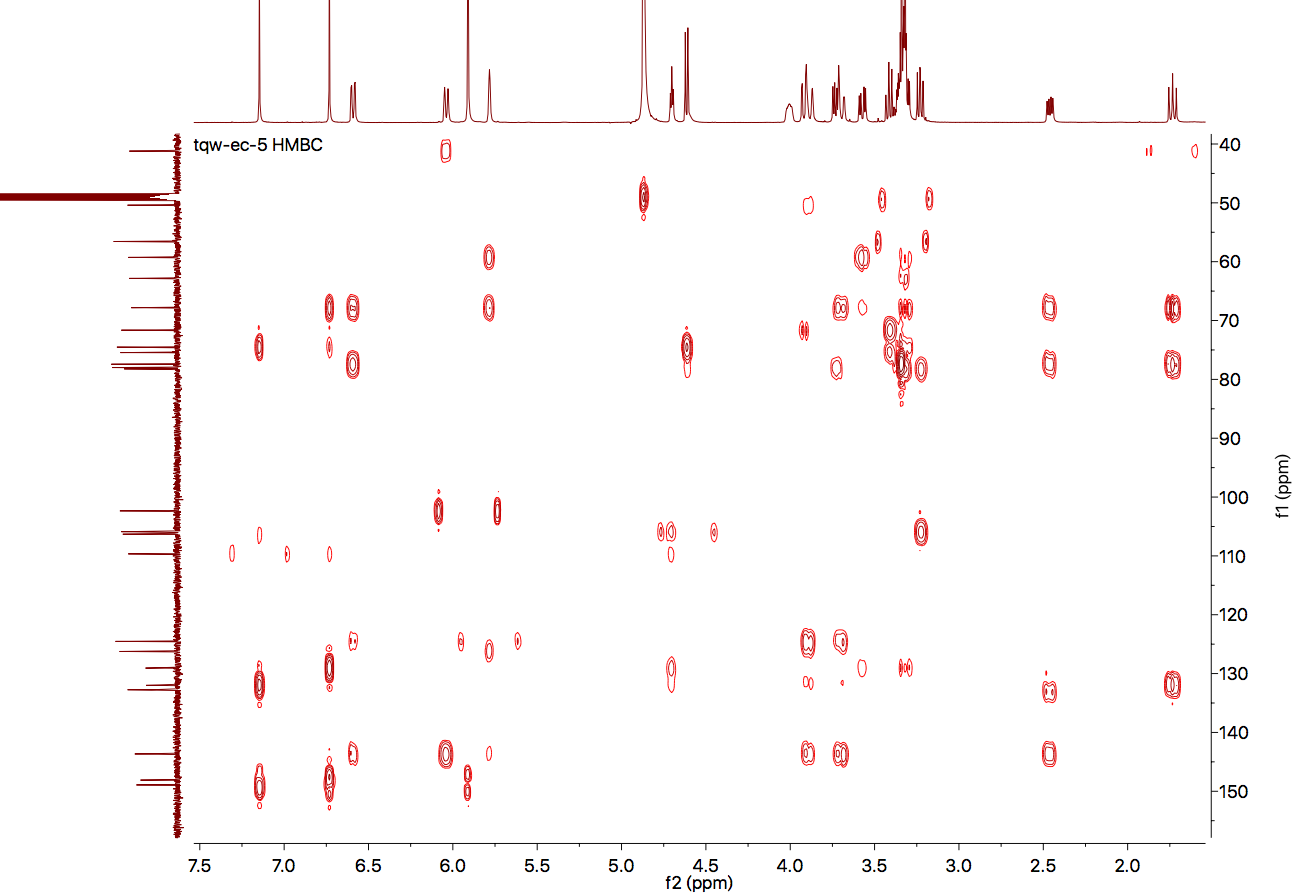
**

**S10. NOESY of erythraline-11*β*-*O*-glucopyranoside (1) in MeOD.**

**
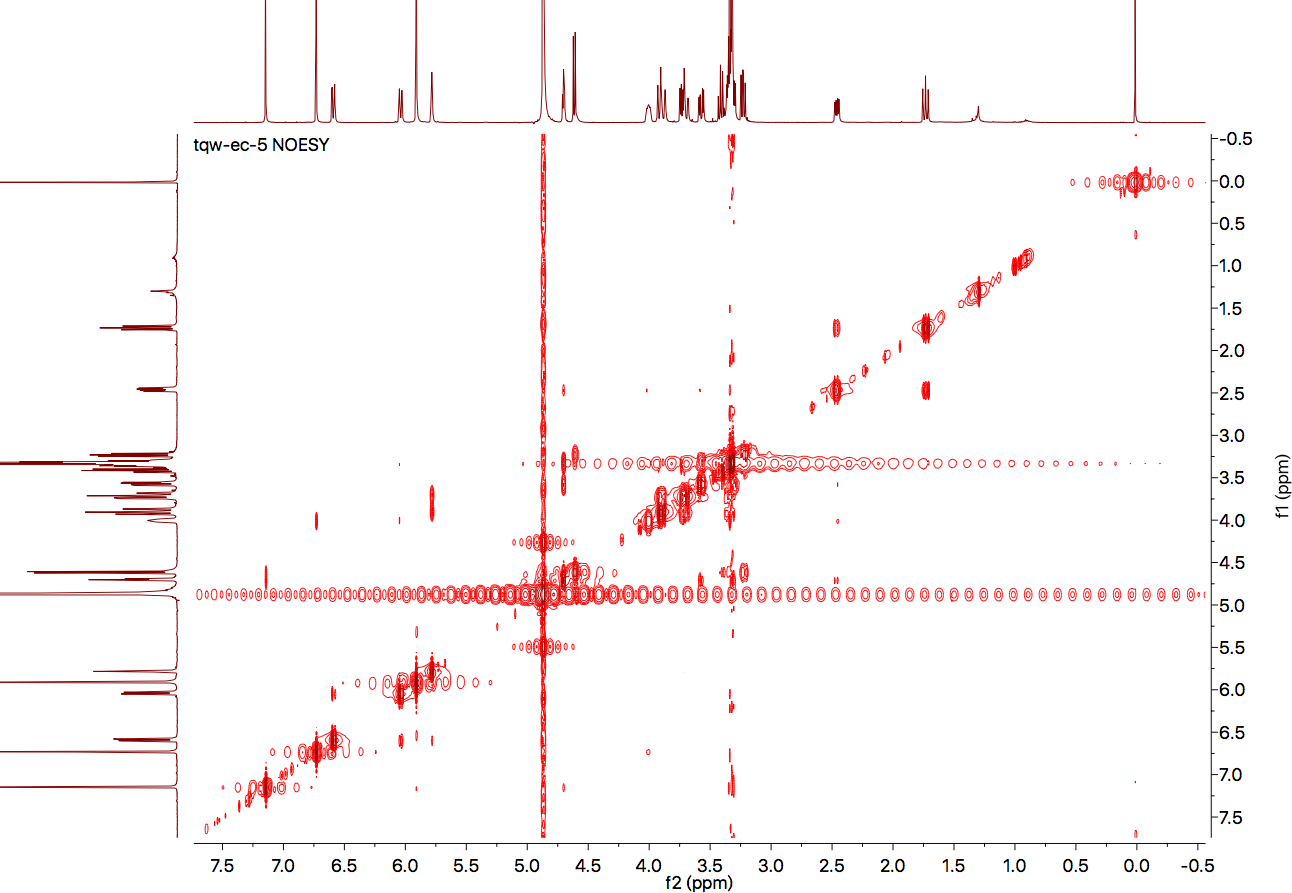
**

**S11. ^1^H NMR spectrum (500 MHz) of erythraline (2) in MeOD.**

**
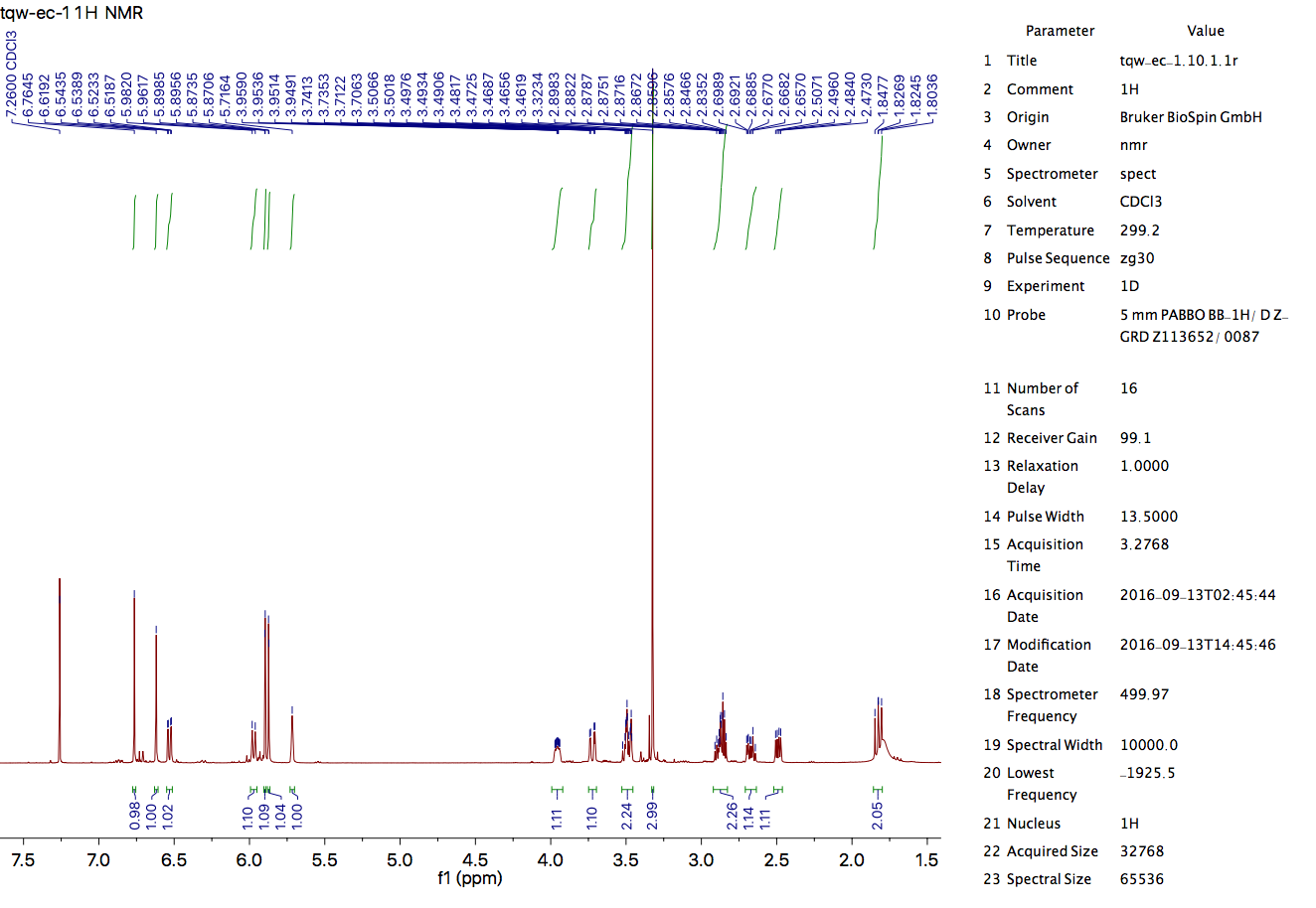
**

**S12. ^13^C NMR spectrum (125 MHz) of erythraline (2) in MeOD.**

**
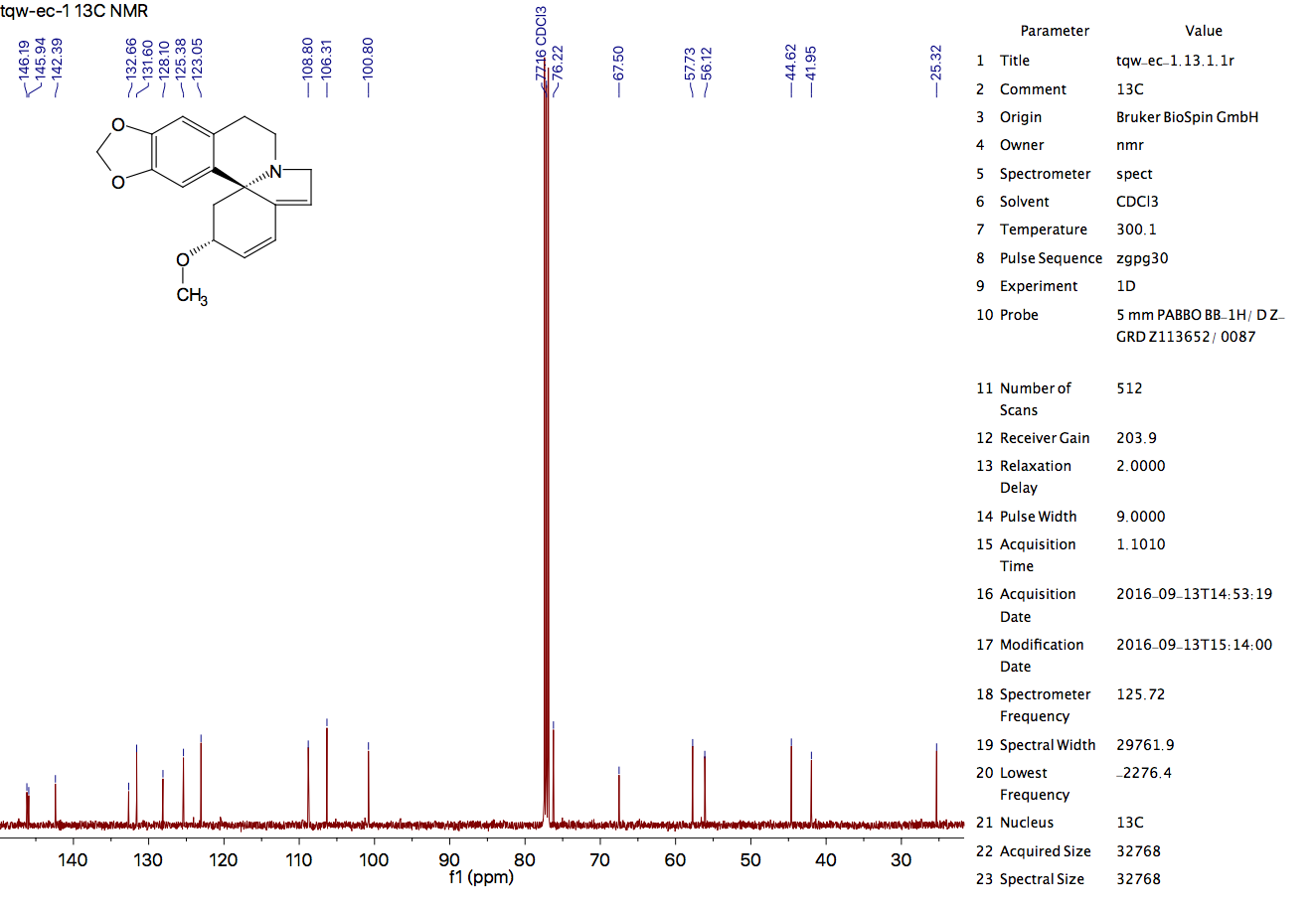
**

**S13. ^1^H NMR spectrum (500 MHz) of erythratine (3) in MeOD.**

**
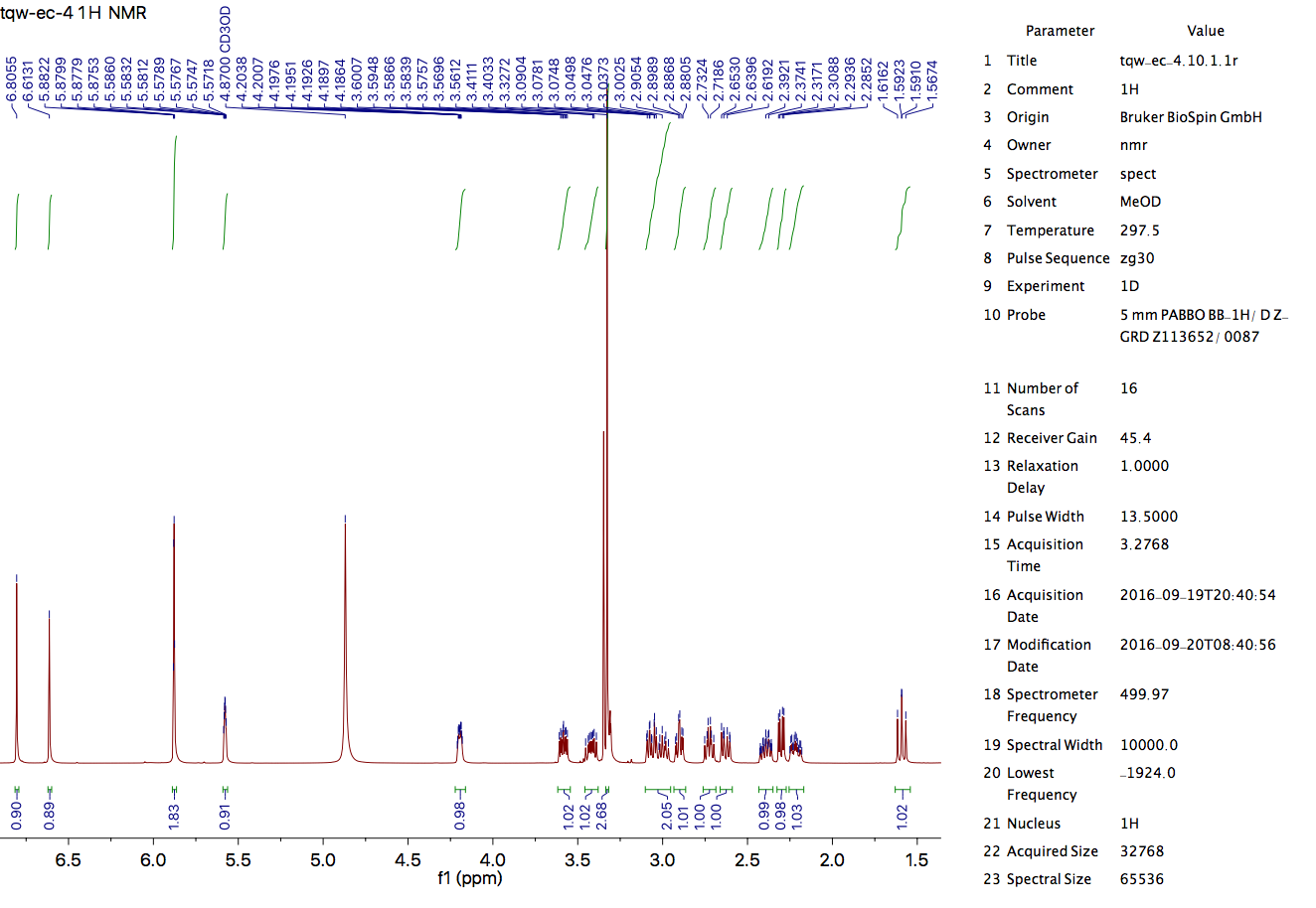
**

**S14. ^13^C NMR spectrum (125 MHz) of erythratine (3) in MeOD.**

**
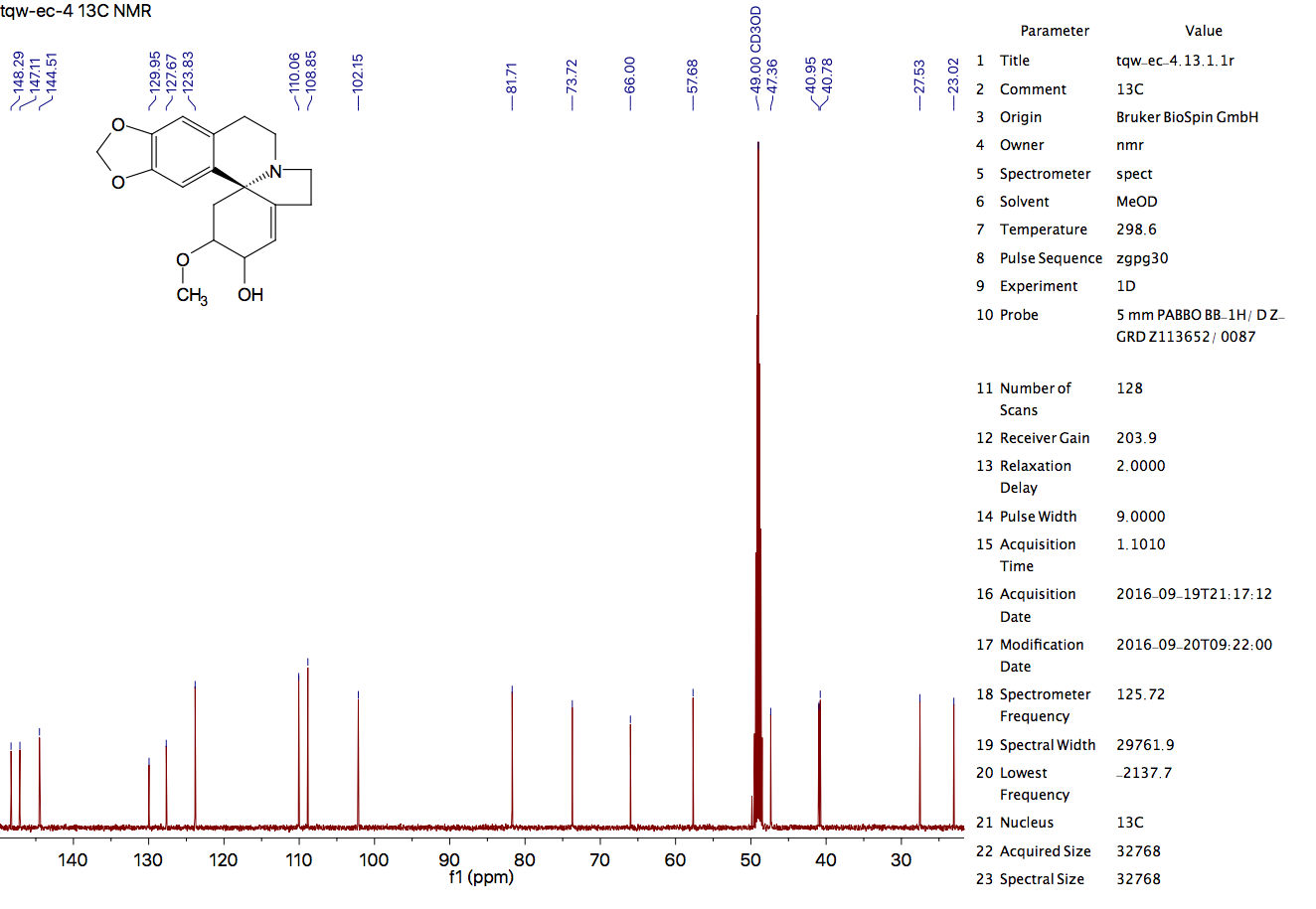
**

**S15. ^1^H NMR spectrum (500 MHz) of erysodine (4) in MeOD.**

**
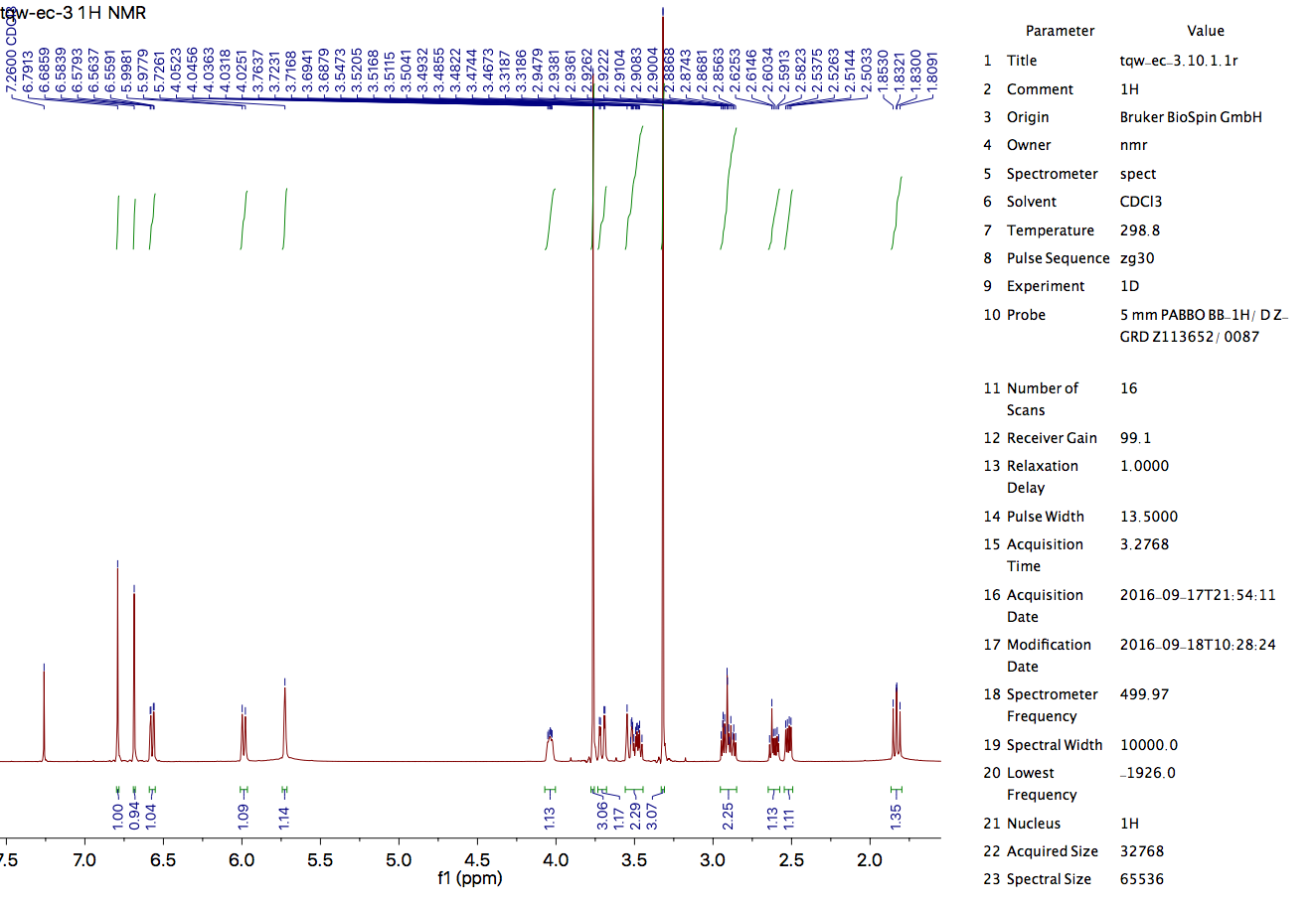
**

**S16. ^13^C NMR spectrum (125 MHz) of erysodine (4) in MeOD.**

**
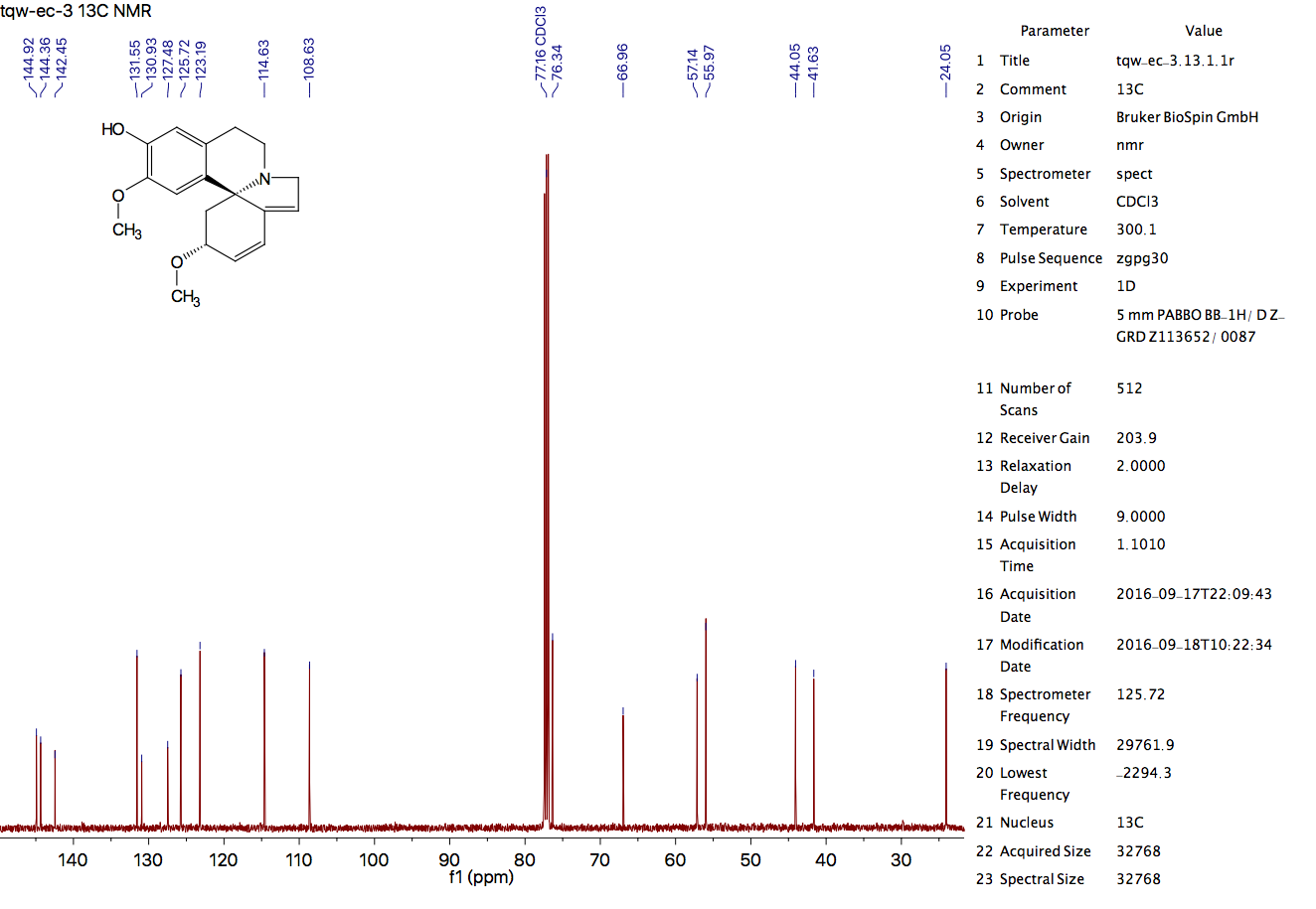
**

**S17. ^1^H NMR spectrum (500 MHz) of erysotrine (5) in MeOD.**

**
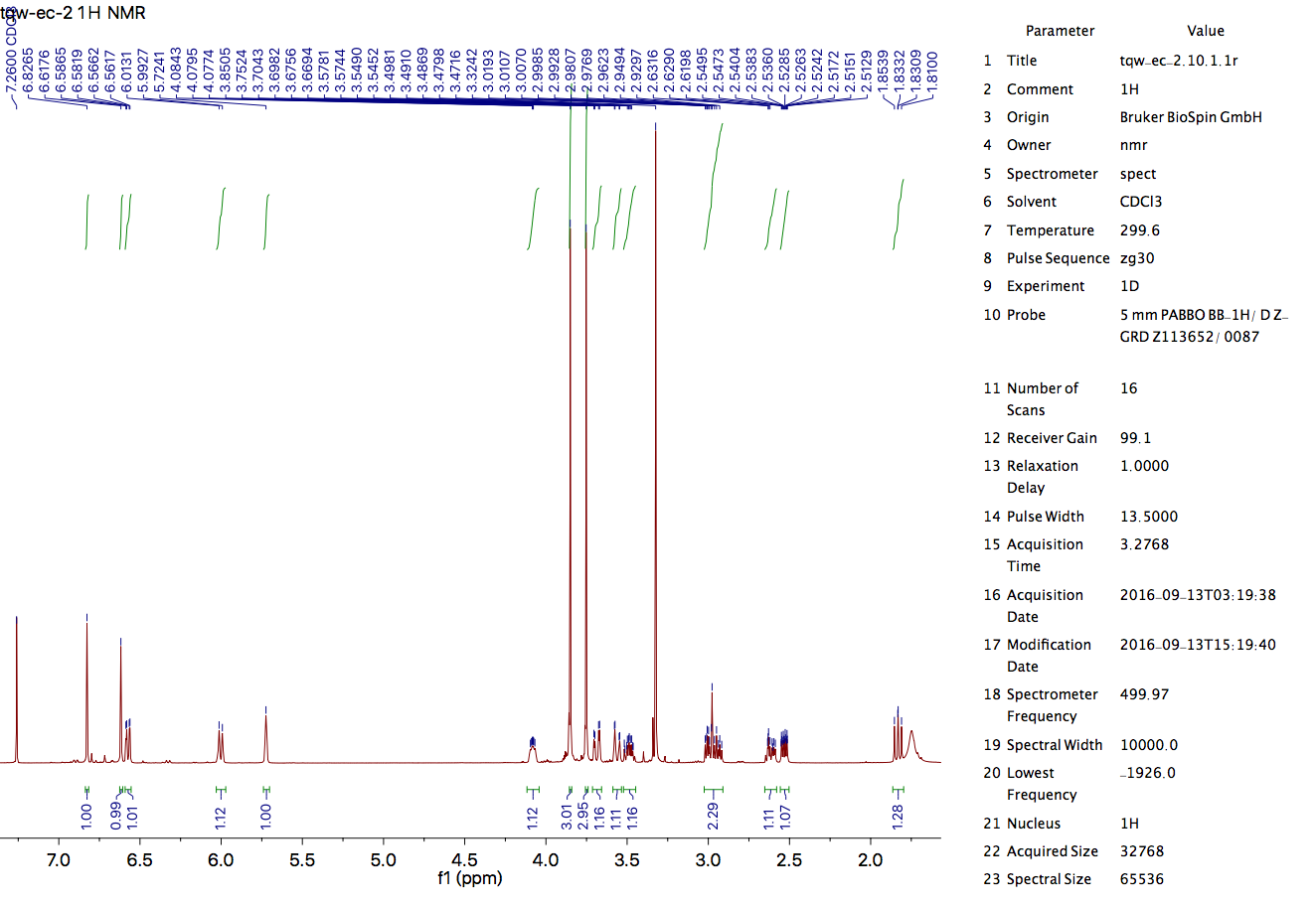
**

**S18. ^13^C NMR spectrum (125 MHz) of erysotrine (5) in MeOD.**

**
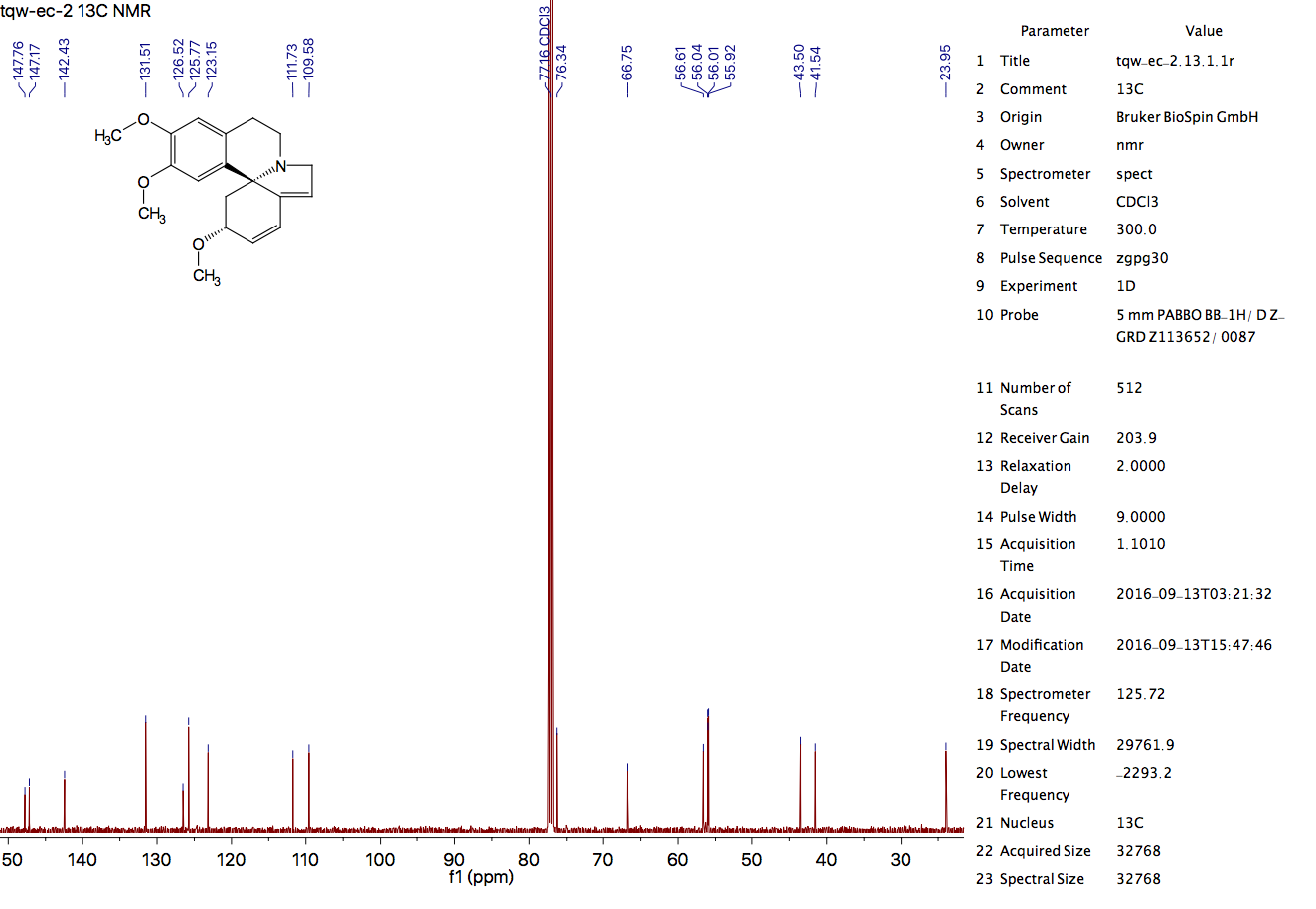
**

**S19. ^1^H NMR spectrum (500 MHz) of (+)-16*β*-D-glucoerysopine (6) in MeOD.**

**
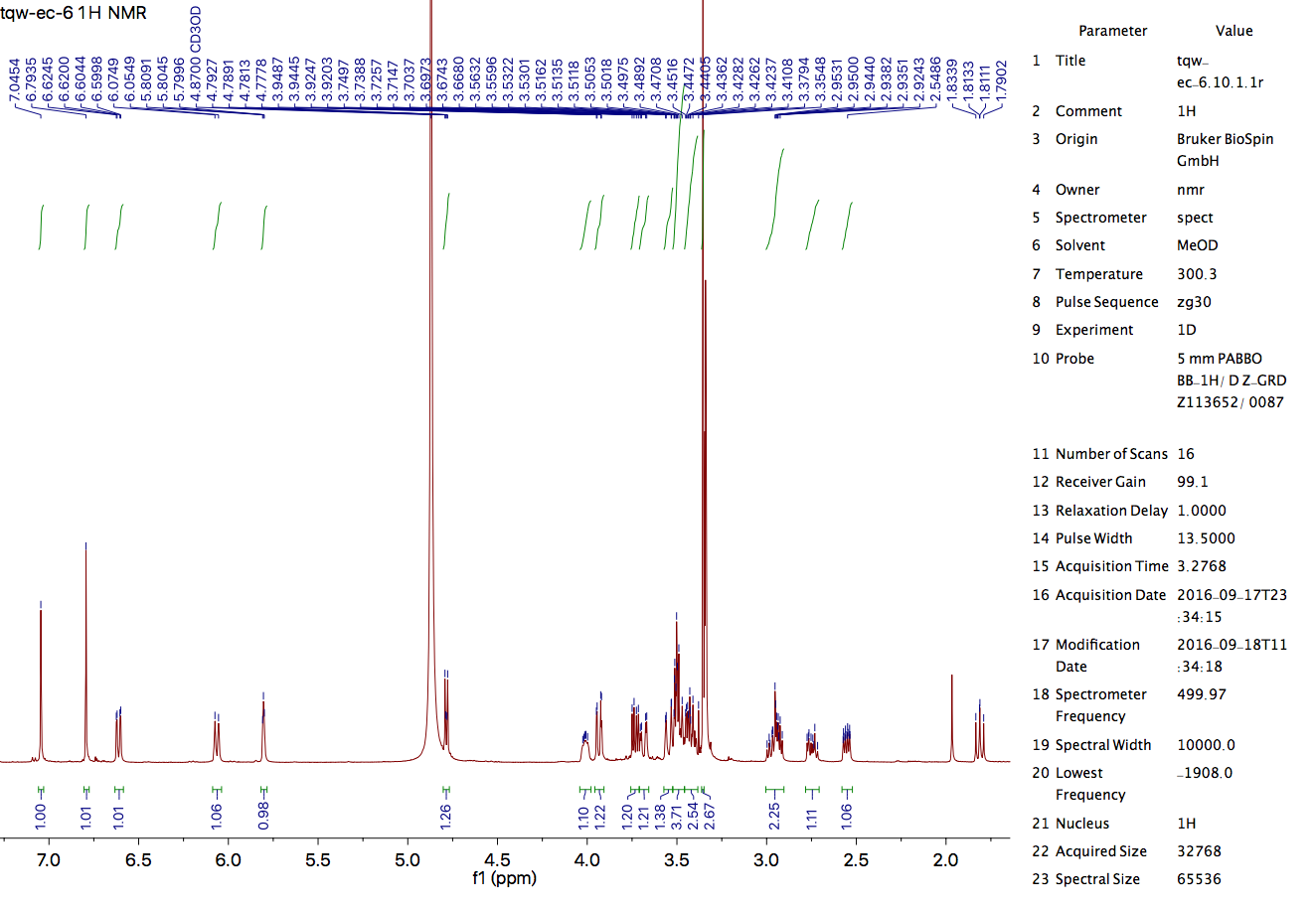
**

**S20. ^13^C NMR spectrum (125 MHz) of (+)-16*β*-D-glucoerysopine (6) in MeOD.**

**
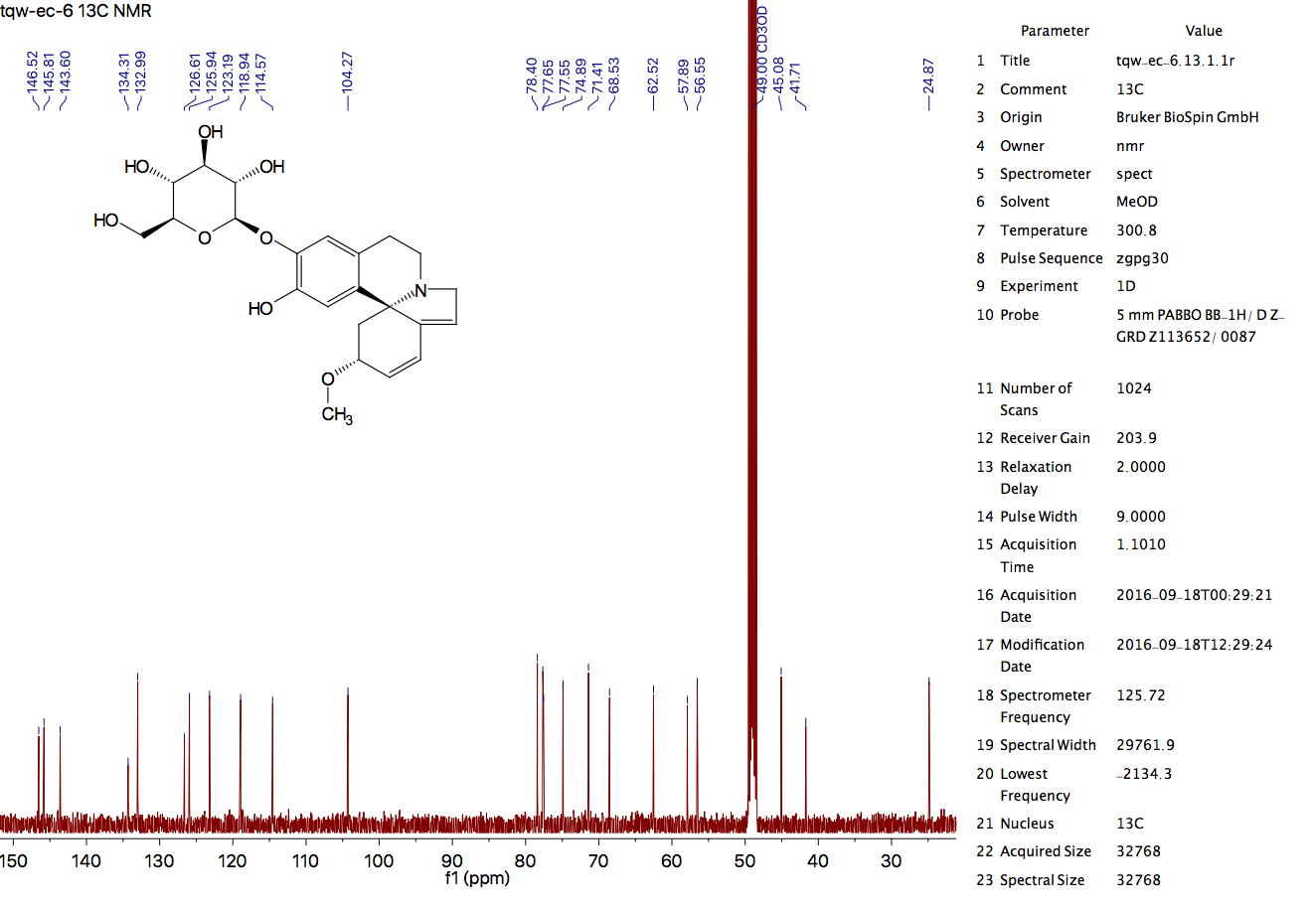
**

**S21. ^1^H NMR spectrum (500 MHz) of (-)-hypaphorine (7) in MeOD.**

**
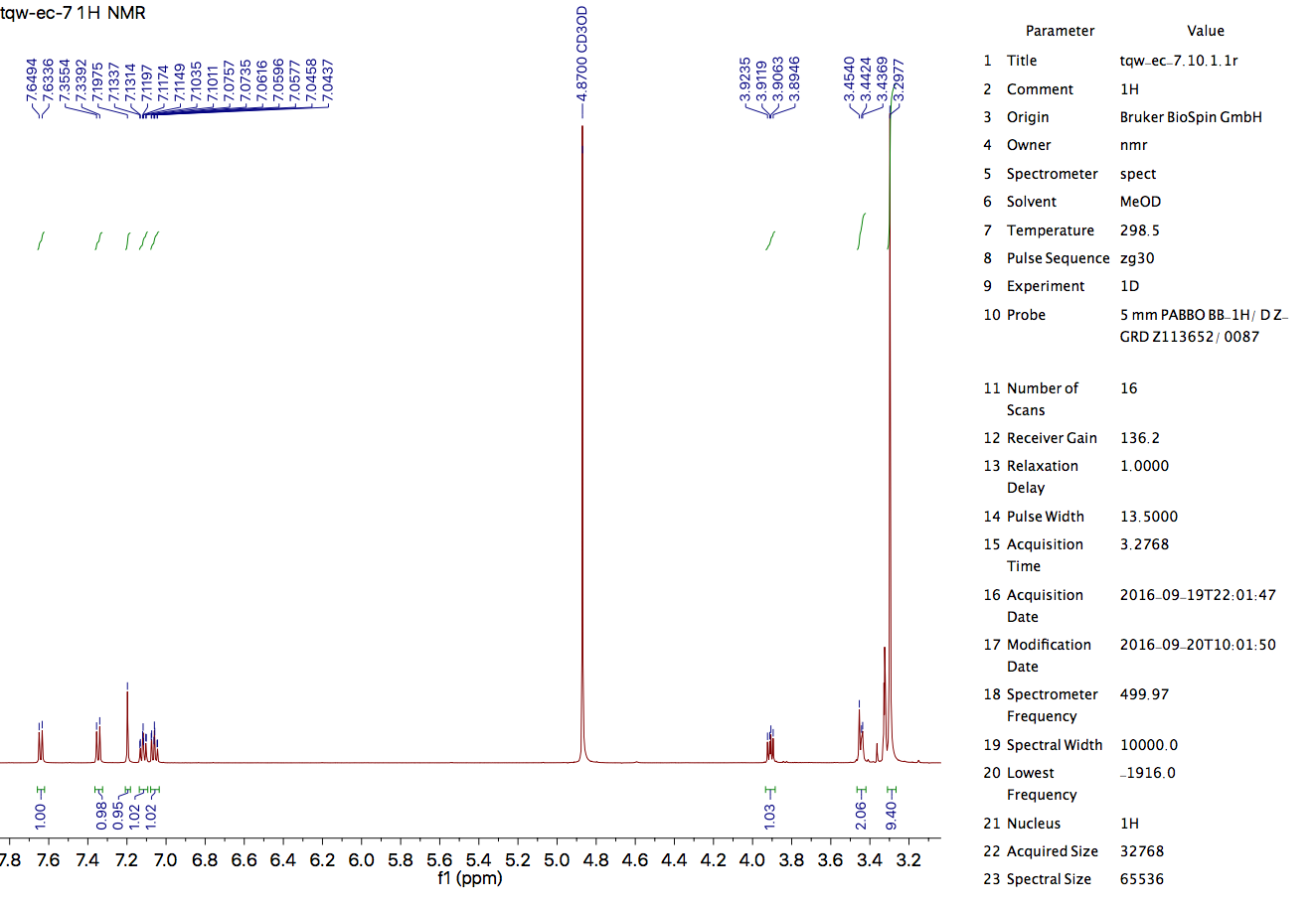
**

**S22. ^13^C NMR spectrum (125 MHz) of (-)-hypaphorine (7) in MeOD.**

**
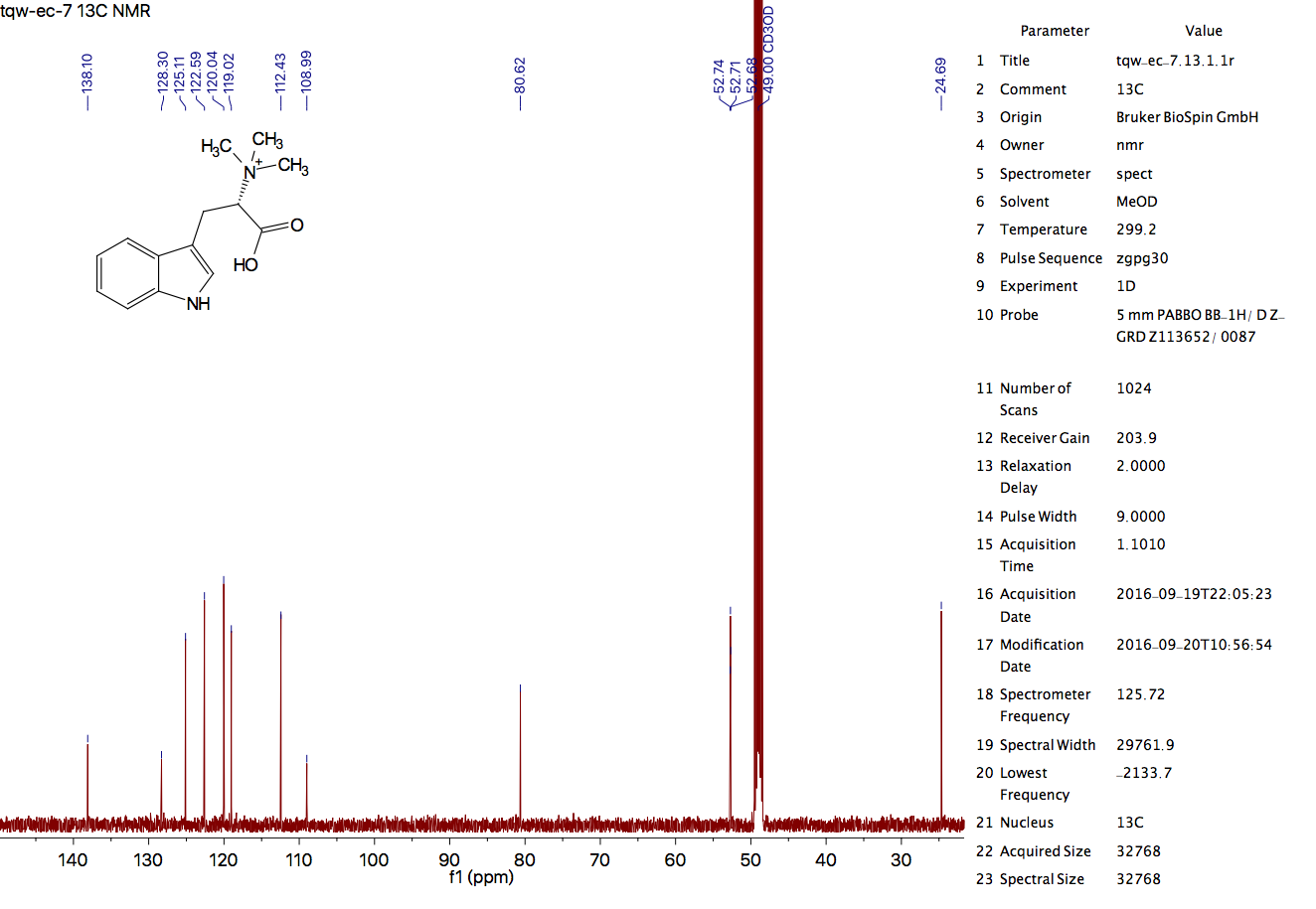
**
